# Impact of Loneliness and Social Support on Acute Health Service Use and Symptom Exacerbation Among Adults with Asthma and COPD

**DOI:** 10.1007/s10880-024-10046-0

**Published:** 2024-10-05

**Authors:** Patric J. Leukel, John D. Piette, Aaron A. Lee

**Affiliations:** 1 Department of Psychology, University of Mississippi, Oxford, MS 38677 USA; 2 Ann Arbor Department of Veterans Affairs Center for Clinical Management Research and Department of Mental Health, Ann Arbor, MI USA; 3 Department of Health Behavior and Health Education, School of Public Health, University of Michigan, Ann Arbor, MI USA

**Keywords:** Asthma, COPD, Loneliness, Social support, Acute health services, Symptom exacerbation

## Abstract

Loneliness and low social support are associated with negative health outcomes among adults with asthma or COPD. Although social support is correlated with loneliness, low social support is neither necessary nor sufficient for the experience of loneliness. This study compares the relative association of loneliness and social support on symptom exacerbation (i.e., acute deteriorations in respiratory health) and acute health service utilization (i.e., hospitalizations, emergency department visits) among 206 adults with asthma and 308 adults with COPD. Separate logistic regression models were used to simultaneously examine the association of loneliness and social support with each outcome. Among adults with asthma, loneliness was associated with greater odds of hospitalization (AOR = 2.81, 95%CI [1.13, 7.02]), while low social support was not (AOR = 1.44, 95%CI [0.78, 2.65]). However, neither loneliness nor social support were associated with any other acute health service use or symptom exacerbation among adults with asthma. Among adults with COPD, loneliness, and greater social support were associated with increased odds of symptom exacerbation (AOR = 1.67, 95%CI [1.03, 2.69]; AOR = 1.36, 95%CI 1.02, 1.83]) and hospitalization (AOR = 3.46, 95%CI [1.65, 7.24]; AOR = 1.92, 95%CI [1.15, 3.22]), but only social support was significantly associated with ED visits (AOR = 1.72, 95%CI 1.12, 2.66]). These findings support prior research demonstrating that loneliness and social support are related but separate determinants of patients’ physical symptoms and service utilization.

Chronic respiratory diseases, such as asthma and chronic obstructive pulmonary disease (COPD) are extremely common and major sources of morbidity, mortality, and acute health services use. In the United States (U.S.) 6.4% of adults have COPD and 8.4% of adults have asthma (CDC, 2020; Xu et al., 2020). COPD is a progressive lung condition characterized by chronic inflammation with obstructed airflow, cough, fatigue, and dyspnea (Lenferink et al., 2018; Reardon et al., 2006). Asthma, on the other hand, is a chronic inflammatory disease which causes cough, wheezing, shortness of breath, and chest tightness (Hammad & Lambrecht, 2021). COPD is the third leading cause of death worldwide and the fourth leading cause of death in the United States (Wheaton et al., 2015). Asthma and COPD are major sources of health care costs and disability (Blanchette et al., 2014; Ivanova et al., 2012). Adults with asthma or COPD are at significantly increased risk of costly emergency department (ED) visits and hospitalizations (Dalal et al., 2011; Hasegawa et al., 2015). Although asthma and COPD have many similarities (e.g., airway inflammation and both tend to lead to symptom exacerbations), there are also several notable differences, such as differences in etiology, symptoms, mediators, response to therapy, and age of onset (Cukic et al., 2012). These differences may contribute to a difference in the way psychological factors, especially social support and loneliness, may impact disease outcomes.

Social support can be generally defined as the tangible or perceived availability of social resources (Holt-Lunstad et al., 2010). There are four commonly accepted forms of social support including tangible (i.e., material assistance), information (e.g., advice, guidance), companionship (e.g., providing a sense of social belonging), and emotional support (i.e., provide reassurance, empathy; Lenferink et al., 2018; Uchino, 2004). Importantly, studies have found self-reported received social support to be only moderately related to perceived social support (Haber et al., 2007; Melrose et al., 2015).

Current research suggests that greater perceived social support is associated with positive health outcomes for adults with asthma and COPD (Barton et al., 2015; Lee et al., 2020; Lind et al., 2015; Ngamvitroj & Kang, 2007). In fact, some evidence suggests that social support is associated with lower risk of developing asthma among adults (Loerbroks et al., 2010). Structural support indicators such as the number of supportive others have been associated with healthy behaviors, such as physical activity and adherence to pulmonary rehabilitation among adults with COPD, as well as medication adherence among individuals with asthma (Chen et al., 2017; Koufopoulos et al., 2016). In contrast, several studies indicate low social support is associated with greater risk of hospitalizations, less effective disease management behaviors, and more frequent symptom exacerbations (i.e., acute deteriorations in respiratory health) among adults with asthma (Metting et al., 2016) and COPD (DiNicola et al., 2013; Lenferink et al., 2018).

Loneliness, which is the subjective feeling of social isolation arising from a discrepancy between perceived and desired quality or quantity of social connection, is often associated with lower perceived social support (Hawkley & Cacioppo, 2010; Letitia Peplau, 1982; Salimi & Bozorgpour, 2012). Yet, low social support is neither a necessary nor sufficient condition for the experience of loneliness (Zhang & Dong, 2022). In support of this conclusion, a recent meta-analysis found an only modest negative correlation (*r* = − 0.39), suggesting that greater social support is protective against feelings of loneliness (Zhang & Dong, 2022).

The prevalence of loneliness among U.S. adults has increased dramatically following the COVID-19 pandemic with recent estimates suggesting that 13.6% of U.S. adults are lonely (Czaja et al., 2021; McGinty et al., 2020). Adults with chronic respiratory disease such as asthma and COPD are at even greater risk of experiencing feelings of loneliness, due to their vulnerability to COVID and the consequent precaution to socially isolate. One recent study found that adults with COPD were three times more likely than adults without COPD to report feeling lonely (Suen et al., 2023). Similarly, another recent study found that adults with asthma reported significantly greater feelings of loneliness compared to healthy controls (Sipowicz et al., 2023).

Like low social support, loneliness is associated with negative health outcomes among adults with chronic respiratory conditions (Agarwal, 2022; Brighton et al., 2022). Both adults with asthma and those with COPD report that the lack of social support and feelings of loneliness are important problems in their lives (Metting et al., 2016). Notably, prior studies have found that loneliness is significantly associated with lower perceived health, and greater risk of ED visits and hospital admissions among adults with chronic respiratory disease (Jordan et al., 2008; Marty et al., 2019). A study of adults with COPD enrolled in a pulmonary rehabilitation program found that loneliness at baseline was associated with lower functional health status and quality of life (Reijnders et al., 2018). The same study found that reductions in loneliness during the pulmonary rehabilitation program were associated with greater improvements in functional exercise capacity and quality of life, suggesting that loneliness may represent an important and potentially modifiable risk factor for poorer respiratory disease outcomes. Another recent study of adults with asthma demonstrated that loneliness is associated with lower meaning in life and poorer asthma control (Sipowicz et al., 2023). Together, these findings suggest that loneliness is a common and potentially important phenomenon among adults with chronic respiratory disease which has been linked to poor respiratory disease control and greater use of acute health services. Although a large body of literature has examined the role of social support in these populations, no studies have simultaneously compared the relative contribution of loneliness and social support on these important chronic respiratory disease related outcomes (i.e., symptom exacerbation, ED visits, and hospitalization) among adults with asthma or COPD. A greater understanding of relative contribution of loneliness and social support to patients’ disease experience through analyses in the same sample is important because such information could point to specific clinical or health services interventions that could improve patients’ quality of life and disease trajectory.

The purpose of the current study was to elucidate the relative associations of loneliness and social support with respiratory symptom exacerbations and acute health service use among adults with asthma and COPD. We hypothesized that loneliness and low social support would be *uniquely* and *independently* associated with greater odds of symptom exacerbation, ED visits, and hospitalizations during the previous 12-month period controlling for their shared variance and for established predictors of respiratory symptom control and use of acute medical services.

## Method

### Participants and Procedures

Participants were recruited from an online panel of adults with chronic respiratory disease maintained Qualtrics in February of 2018. All panel members had previously reported having a chronic respiratory disease including asthma and/or COPD. Specifically, these panel members are contacted by Qualtrics and recruited to participate in research studies in exchange for monetary compensation. To be eligible, panel members had to be over the age of 18, located in the United States, and report having been diagnosed with asthma *or* COPD. Eligible participants provided informed consent and completed a battery of self-report measures. A total of 913 individuals initiated the survey, 840 (90%) of which provided informed consent. Of those who provided informed consent, 230 reported having asthma only, 321 reported having COPD only, and 217 reported having both asthma and COPD. Individuals who reported having been diagnosed with both asthma and COPD (*n* = 217) were excluded from data analysis. This step was taken to avoid the confounding of results across the disease groups. In total, 206 (90%) among those with asthma, as well as 308 (96%) among those with COPD completed all study measures and were therefore included in the final sample. All study procedures were approved by the University of Mississippi Institutional Review Board.

## Measures

### Symptom Exacerbations

Acute symptom exacerbations were defined as a period of progressively worsening respiratory symptoms beyond normal daily variability in symptoms. Using and providing this definition, participants were asked to report how many symptom exacerbations they experienced in the past 12 months (“How many symptom exacerbations have you had in the past 12 months?”). Participant responses were dichotomized (no exacerbation = 0, $\ge$ 1 symptom exacerbation = 1). At least one prior study indicates that adults with COPD are relatively accurate in reporting their symptom exacerbations during the prior 12-month period (Quint et al., 2011).

### Use of Acute Medical Care

ED visits were measured by asking respondents if they had visited an emergency department in the past twelve months (“Have you visited an emergency room in the past 12 months because of your respiratory condition?”; “No” = 0, “Yes” = 1). Similarly, respondents were asked whether they had to be admitted to the hospital in the past twelve months due to their respiratory condition (“Have you been admitted to the hospital in the past 12 months because of your respiratory condition?”; “No” = 0*,* “Yes” = 1). Prior studies suggest that adults are accurate in recalling ED visits and hospitalizations—which tend to be high (Bhandari & Wagner, 2006; Iversen et al., 2007; Petrou et al., 2002).

### Loneliness

The 3-item version of the UCLA Loneliness Scale was used to measure participants’ subjective perception of loneliness (Hughes et al., 2004). The scale consists of three items that each measure a dimension of loneliness: relational connectedness (“How often do you feel that you lack companionship?”), social connectedness (“How often do you feel left out?”), and self-perceived isolation (“How often do you feel isolated from others?”). Participants rate each item on a Likert-type scale from 1 (“Hardly ever”) to 3 (“Often”). Item responses are averaged to generate a total score ranging from 1 to 3, with higher scores indicating greater loneliness. The UCLA 3-Item Loneliness Scale has demonstrated adequate internal consistency (*α* = 0.72) in previous samples of adults (Hughes et al., 2004) and strong internal consistency among the current samples of adults with asthma (*α* = 0.83) and COPD (*α* = 0.87). The scale also has good concurrent and discriminant validity, and good convergent validity with longer measures of loneliness (Hughes et al., 2004).

### Social Support

The ENRICHD Social Support Inventory (ESSI) was used to measure participants’ level of perceived and functional social support (Mitchell et al., 2003). The self-report measure consists of seven items, which address emotional (four questions), informational (one question), and practical (one question) social support, as well as one question asking about the presence of a romantic partner. Participants rate six of the items on a Likert scale from 1 (“none of the time”) to 5 (“all of the time”). The last item about the romantic partner is answered on a dichotomous scale with 1 (“Yes”) and 2 (“No”), with a positive response being worth four points and a negative response worth two points. Item responses were used to calculate a mean score ranging from 1 to 5, with higher scores indicating greater social support. The ESSI has demonstrated good internal consistency (*α* = 0.86) in a previous sample consisting of patients recovering from myocardial infarction (Mitchell et al., 2003). It has also demonstrated good internal consistency among adults with asthma (*α* = 0.88) and COPD (*α* = 0.88) in the current sample. The scale has demonstrated convergent validity with similar measures of perceived social support, whereas discriminant validity has been demonstrated with social support measures that are focused on received and structural social support (Mitchell et al., 2003).

### Control Variables

Both loneliness and social support can vary substantially across sociodemographic groups that differ in their disease trajectory and health service use. The current study controlled for age, sex, race, ethnicity (“Are you of Hispanic or Latino origin?”), number of primary care visits (“How many visits have you had to your primary care provider in the past 12 months?”), and smoking status (“Do you now smoke cigarettes every day, some days, or not at all?”) for all data analyses. The number of primary care visits during the past 12 months was included as a control variable as one or more primary care visits per year has been found to be associated with increased likelihood of improved long-term health outcomes due to preventative care interventions (Hostetter et al., 2020). Moreover, both COPD and adult asthma are recognized as ambulatory care sensitive conditions for which primary care engagement is believed to reduce potentially preventable emergency department visits and hospitalizations (*AHRQ QI: Emergency Department Prevention Quality Indicators Overview*, n.d.; Loyd et al., 2023) Additionally, smoking status was entered as a control variable as smoking has been established as one of the strongest predictors of early mortality and symptom exacerbation among individuals with chronic respiratory diseases (Polosa & Thomson, 2013; Sethi & Rochester, 2000). Finally, we controlled for potentially important demographic characteristics including age, sex (male = 0, female = 1), race (White = 0, Other = 1), and ethnicity (Hispanic = 0; non-Hispanic = 1).

### Data Analysis

All analyses were performed using SPSS version 28. Descriptive statistics were used to characterize the samples of adults with asthma and COPD. Binary logistic regression models were used to examine the association between the UCLA-3 and ESSI with each outcome (i.e., symptom exacerbation, ED visits, hospitalization) separately among the samples of adults with asthma and COPD. Each model controlled for established predictors of study outcomes including age, sex, race, ethnicity, number of primary care visits, and smoking status (Burney et al., 2015; Chowdhury et al., 2021; Eisner et al., 2011; Hughes et al., 2017; Laniado-Laborín, 2009; Polosa & Thomson, 2013; Price et al., 2011; Talreja & Baptist, 2011; Yawn, 2011). Control variables were entered in step one. UCLA-3 and ESSI were added in step two. All inferential statistical analyses were two-tailed with alpha = 0.05.

## Results

### Sample Characteristics

The combined sample of adults with asthma and COPD was approximately half female (50%) and predominantly White (70%) with an average age of 53 years (Table 1). Most participants had either public or private health insurance (95%) and most participants had a primary care provider (95%) within the past 12 months. Approximately half of the sample were current smokers (46%). Many participants presented to the ED (19%) or were hospitalized (13%) at least once in the prior 12 months for respiratory symptoms. Nearly two thirds of participants experienced symptom exacerbation in the prior 12 months (61%).

**Table 1:** Participant characteristics (*N* = 551)

	Overall (*N* = 551)	Asthma (*n* = 230)	COPD (*n* = 321)
	% (*n*)	% (*n*)	% (*n*)
Age^a^	53.1(17.44)	41.9 (16.00)	61.5 (14.89)
Female	50.1 (276)	33.0 (76)	62.0 (199)
*Race*			
White	70.2 (387)	44.8 (103)	88.5 (284)
Black	16.5 (91)	28.7 (66)	7.8 (25)
Asian	7.4 (41)	14.8 (34)	2.2 (7)
American Indian	2.4 (13)	3.5 (8)	1.6 (5)
Hispanic	17.6 (97)	31.7 (73)	7.5 (24)
Health insurance^b^	94.6 (521)	91.7 (211)	96.6 (310)
Has primary care provider^b^	94.7 (522)	93.0 (214)	96.0 (308)
# of primary care visits^abc^	3.01 (1.33)	2.85 (1.27)	3.12 (1.35)
Current smoker	45.7 (252)	43.5 (100)	47.4 (152)
Symptom exacerbation^ab^	0.61 (0.49)	0.63 (0.48)	0.60 (0.49)
Emergency department visit^ab^	0.19 (0.39)	0.19 (0.39)	0.19 (0.39)
Hospitalization^ab^	0.13 (0.34)	0.13 (0.34)	0.13 (0.34)
ESSI^a^	3.55 (1.02)	3.60 (0.94)	3.51 (1.06)
UCLA Loneliness Questionnaire^a^	1.77 (0.66)	1.81 (0.64)	1.74 (0.67)

^a^Mean (*SD*)

^b^Past 12 months

^c^Only assessed among respondents who reported having a primary care provider

*ESSI* ENRICHD Social Support Inventory

### Asthma

Among adults with asthma, loneliness was significantly and independently associated with greater odds of hospitalization (*p* = 0.027; see Table 2) but not with symptom exacerbation (*p* = 0.424) or ED visits (*p* = 0.082) after controlling for social support and patient characteristics (i.e., age, sex, race, ethnicity, number of primary care visits during the prior 12 months, and smoking status). Social support was not significantly associated with odds of symptom exacerbation (*p* = 0.864), ED visits (*p* = 0.963), hospitalization (*p* = 0.247) in the prior 12 months controlling for loneliness and patient characteristics.

**Table 2:** Results of adjusted logistic regression models examining the association between loneliness and social support with symptom exacerbation and acute health care use among adults with asthma (*n* = 206)

	Acute symptom exacerbation			ED visits			Hospitalization		
	AOR	SE	95%CI	AOR	SE	95%CI	AOR	SE	95%CI
*Step 1*									
Age	0.40	.01	0.97, 1.01	0.97	.02	0.94, 1.00	0.95	.02	0.90, 0.99
Sex	0.61	.33	0.44, 1.62	0.45	.41	0.20, 1.02	0.26	.51	0.09, 0.70
Race	0.94	.32	0.52, 1.82	1.60	.43	0.69, 3.70	1.68	.55	0.57, 4.98
Ethnicity	0.22	.36	0.32, 1.30	0.65	.41	0.29, 1.45	0.61	.51	0.23, 1.64
Primary care visits	1.29	.13	1.01, 1.65	1.10	.17	0.80, 1.53	1.02	.21	0.67, 1.54
Current smoker	2.52	.34	1.29, 4.93	2.32	.43	1.00, 5.37	5.79	.62	1.73, 19.40
*Step 2*									
ESSI	1.03	.20	0.70, 1.52	0.99	.24	0.62, 1.57	1.44	.31	0.78, 2.65
UCLA Loneliness Questionnaire	1.26	.29	0.72, 2.22	1.87	.36	0.92, 3.80	2.81	.47	1.13, 7.02

*ESSI* ENRICHD Social Support Inventory

### COPD

Among adults with COPD, loneliness was significantly associated with greater odds of symptom exacerbation (*p* = 0.036; see Table 3) and hospitalization (*p* = 0.001), but not ED visits (*p* = 0.050) after controlling for social support and patient characteristics (i.e., age, sex, race, ethnicity, number of primary care visits during the prior 12 months, and smoking status). Greater social support was significantly associated with greater odds of symptom exacerbation (*p* = 0.039), ED visits (*p* = 0.014), and hospitalization (*p* = 0.013) in the prior 12 months after controlling for loneliness and other patient characteristics.

**Table 3:** Results of adjusted logistic regression models examining the association between loneliness and social support with symptom exacerbation and acute health care use among adults with COPD (*n* = 308)

	Acute symptom exacerbation			ED visits			Hospitalization		
	AOR	SE	95%CI	AOR	SE	95%CI	AOR	SE	95%CI
*Step 1*									
Age	0.97	.01	0.94, 0.99	0.98	.01	0.95, 1.01	0.99	.02	0.96, 1.02
Sex	1.07	.27	0.63, 1.82	1.37	.34	0.70, 2.65	0.81	.41	0.36, 1.82
Race	1.53	.57	0.51, 4.62	1.29	.53	0.46, 3.62	0.74	.65	0.21, 2.66
Ethnicity	0.19	1.10	0.02, 1.65	0.27	.63	0.08, 0.91	0.15	.71	0.04, 0.58
Primary care visits	1.24	.10	1.03, 1.50	1.43	.13	1.12, 1.84	1.41	.15	1.05, 1.89
Current smoker	1.06	.27	0.63, 1.78	1.30	.35	0.66, 2.57	1.88	.43	0.82, 4.32
*Step 2*									
ESSI	1.36	.15	1.02, 1.83	1.72	.22	1.12, 2.66	1.92	.26	1.15, 3.22
UCLA Loneliness Questionnaire	1.67	.24	1.03, 2.69	1.86	.32	1.00, 3.44	3.46	.38	1.65, 7.24

*ESSI* ENRICHD Social Support Inventory

## Discussion

This study is the first to simultaneously examine differences in the associations between loneliness and social support as predictors of symptom exacerbations and acute health service use among adults with asthma or COPD. Our results suggest that loneliness and social support have different but often aligned impacts on acute health service use (i.e., ED visits, and hospitalizations)—even after accounting for relevant patient risk factors among adults with COPD. However, among adults with asthma loneliness appears to carry a stronger risk for negative health outcomes compared to social support.

### Asthma

As predicted, loneliness was significantly associated with risk of hospitalization among adults with asthma such that each one unit increase in the UCLA loneliness scale (range: 1 to 3) was associated with nearly threefold increase in risk of self-reported hospitalization during the previous year. This finding is in line with results from at least one prior study showing that loneliness is associated with poorer asthma symptom control (Sipowicz et al., 2023). However, contrary to prediction, loneliness was not significantly associated with symptom exacerbation or ED visits among adults with asthma. These findings were unexpected as exacerbations and ED visits are often a precursor to hospitalization among individuals with asthma (Bousquet et al., 2005). Viewed in the context of the broader literature, these findings suggest loneliness may be a vulnerability for acute health care utilization among adults with asthma.

Contrary to our hypothesis, social support was not associated with symptom exacerbations, ED visits, or hospitalization among adults with asthma. These null findings were unexpected in light of evidence from previous studies linking low social support with poorer asthma outcomes. For example, one prior study found lower perceived social support was associated with low medication adherence in a group of adolescents with asthma, indicating poor self-management in the absence of perceived social support (Sloand et al., 2021). However, this study did not simultaneously examine the impact of loneliness. Another study found greater perceived social support to be associated with less negative mood, lower stress severity, and fewer asthma-specific symptoms (i.e., wheezing and coughing) among adults with asthma (Smyth et al., 2014). One study even found social support was inversely related to risk of adult asthma (Loerbroks et al., 2010).

This discrepancy in findings may be attributable to differences in study methods. First, previous studies examined the link between social support and more proximal asthma related outcomes (e.g., self-management behaviors, asthma symptom control), whereas the present study examined the relationship of social support with more distal outcomes (symptom exacerbations, respiratory related ED visits and hospitalizations). It is possible that social support facilitates improvements in proximal disease outcomes but has weaker impact on more distal outcomes. Second, unlike previous studies, the current study controlled the shared variance between social support and loneliness which may have attenuated the observed relationship between social support and our study outcomes. Third, most studies examining social support among individuals with asthma have focused on children or adolescents, rather than adults. This presents a unique challenge as the effects of perceived social support may differ significantly between age groups. Indeed, one recent study found social support was associated with poorer asthma control among older adults with asthma (Greenfield et al., 2023). Neither this finding, nor those of the current study provide direct evidence for beneficial effects of social support on respiratory outcomes among adults with asthma. Indeed, the present findings suggest that, compared to social support, loneliness may play a more important role in risk of hospitalizations among adults with asthma.

### COPD

Consistent with prediction, loneliness was associated with greater risk of symptom exacerbations and hospitalizations among adults with COPD. The association between loneliness and ED visits approached but did not reach statistical significance. Overall, these findings are generally consistent with previous literature demonstrating that loneliness and social isolation are linked with greater respiratory related ED visits and hospitalizations among adults with COPD (Jordan et al., 2008; Marty et al., 2019). However, this study is the first to demonstrate that loneliness is independently associated with increased risk of acute service among adults with COPD after controlling for social support. Viewed in the context of the broader literature, findings from this study suggest that loneliness represents a particularly important risk factor for symptom exacerbations and acute health service use among adults with COPD. The importance of these findings is underscored by findings from previous studies showing that loneliness is substantially more common among adults with vs. without COPD (Suen et al., 2023).

Contrary to prediction, greater social support was associated with *greater* odds of symptom exacerbation, ED visits, and hospitalizations among adults with COPD after controlling for loneliness and patient’s sociodemographic and other characteristics. This result was unexpected given the large body of research linking social support to more favorable outcomes among adults with COPD, such as improved self-efficacy and medication adherence (Barton et al., 2015; Bonsaksen et al., 2012; Kara Kaşıkçı & Alberto, 2007). As noted previously, at least one prior study found a seemingly paradoxical relationship between greater social support and poorer symptom control among adults with asthma. We suggest two possibilities to explain these seemingly paradoxical findings. First, it is likely that family members and friends provide more support to individuals with more severe respiratory symptoms who are also more likely to experience symptom exacerbations and use acute medical services. Second, these supporters may facilitate their support recipients’ use of emergency medical care which may subsequently increase their likelihood of hospitalizations. For example, patients who receive more support may be more likely to have family or friends who encourage them to seek medical treatment and/or provide transportation to emergency medical services. Additional studies are needed to corroborate these findings and identify confounding or mediating variables that may explain the observed link between greater social support and increased risk of symptom exacerbations and hospitalization among adults with COPD.

## Limitations

The results of this study should be interpreted in the context of several notable limitations. First, the current study relied on participants’ self-report of their chronic respiratory disease diagnosis as well as their recall of symptom exacerbations and respiratory disease related acute service use (i.e., ED visits and hospitalizations) during the prior 12 months. However, current research suggests individuals accurately self-report their chronic respiratory diagnosis as well use of acute medical service during the previous year (Bhandari & Wagner, 2006; Iversen et al., 2007; Petrou et al., 2002). Second, we did not assess the frequency of respondents’ symptom exacerbations, ED visits, or hospitalizations during the prior year. Such information would provide more precise estimates of the risk associated with loneliness and social support. To address these limitations, additional studies could use medical record data to examine the incidence of each outcome associated with loneliness and social support. Third, the observational nature of this study precludes causal inference among study variables. For example, adults who experience more frequent symptom exacerbations may receive more social support from concerned family members and friends. Fourth, data was collected prior to the COVID-19 pandemic and therefore may underestimate the prevalence of loneliness among respondents with asthma or COPD. For example, social distancing may limit access to social support and exacerbate the negative impact of loneliness. Fifth, for methodologic clarity we excluded a relatively large number of survey respondents who reported both asthma and COPD, and their experience of their pulmonary disease and its relationship to loneliness or isolation may be different than that of people who do not experience that multi-morbidity. Sixth, although the present study highlights the importance of loneliness and low support as risk factors for poor respiratory disease outcomes, we are unable make specific recommendations about the identification of patients at risk for loneliness or low social support. Finally, we are not able to account for respondents’ adherence to long or short acting respiratory medications. It is possible that social support reduces risk of negative respiratory disease outcomes (e.g., exacerbations, acute health care use) via improvements in medication adherence.

### Clinical Implications

Hospitalizations or ED visits are associated with increased risk of contracting nosocomial (i.e., a disease originating in a hospital) respiratory diseases, which are particularly harmful to adults with chronic respiratory diseases, such as asthma and COPD (Boukhris et al., 2020). Prior studies have found that adults with asthma and COPD are more likely to contract COVID-19 and when infected, experience greater respiratory distress, more significant negative health outcomes, and risk of death (Adir et al., 2021; Gerayeli et al., 2021). During the pandemic, the CDC recommended socially distancing to reduce risk of contracting coronavirus. However, social distancing practices may contribute to even greater loneliness and limited social support among this already vulnerable group of adults (Suen et al., 2023). Additional work is needed to develop and refine methods to screen and identifying adults with chronic respiratory disease at increased risk for loneliness and low social support.

The differences in experiences of symptoms between asthma and COPD may have contributed to these surprising findings. Whereas individuals with asthma tend to experience more acute symptoms, such as asthma attacks, adults with COPD suffer from more constant symptoms, such as chronic dyspnea (Anzueto & Miravitlles, 2017; Welte & Groneberg, 2006). Given the long-term suffering involved among those with COPD, feelings of loneliness may grow more meaningful over time, whereas those with asthma may be less impacted given the acute nature of the symptomology. Future studies should seek to explore these dynamics, taking the acuteness of each illness into account.

The results of the current study suggest that loneliness and social support carry different risks for symptom exacerbations and acute medical service among adults with asthma and COPD. Loneliness appears to be a more important risk factor among adults with COPD than those with asthma. The present findings suggest that interventions to enhance social support among adults with chronic respiratory disease may be ineffective if they fail to address loneliness. Prior research suggests that increasing social connectedness may be a promising approach to reduce loneliness. For example, interventions which focus on purposeful social activities have been shown to decrease loneliness (O’Rourke et al., 2018). Considering such interventions may play a pivotal role in not only increasing social support opportunities but also simultaneously diminishing loneliness.

## Data Availability

Data and code from the current study will be made available to individuals upon request.
